# Peripheral immune-inflammatory markers associated with intracranial progression-free survival after Gamma Knife radiosurgery in patients with non-small cell lung cancer brain metastases: a retrospective cohort study

**DOI:** 10.3389/fmed.2026.1941822

**Published:** 2026-10-13

**Authors:** Wei Han, Hualin Wei, Ting Zeng, Xiangyong Yue, Xianbo Zhang, Jing Zhao

**Affiliations:** 1 Department of Oncology, Hebei General Hospital, Shijiazhuang, China; 2 Department of Oncology, The First People’s Hospital of Yibin, Yibin, China

**Keywords:** brain metastases, Gamma Knife radiosurgery, immune-inflammatory markers, non-small cell lung cancer, prognostic model, progression-free survival

## Abstract

**Background:**

Intracranial outcomes after Gamma Knife radiosurgery (GKRS) for non-small cell lung cancer (NSCLC) brain metastases vary substantially. Peripheral immune-inflammatory markers may provide additional prognostic information, but their associations with composite intracranial progression-free survival (iPFS) and with documented intracranial progression remain unclear. We therefore evaluated these two outcomes separately.

**Methods:**

We retrospectively analyzed 104 patients with NSCLC brain metastases treated with GKRS between July 2019 and June 2021. Composite iPFS was defined from GKRS to intracranial progression or death from any cause. Documented intracranial progression was additionally analyzed with death before progression treated as a competing event. Six covariates, predefined before fitting the primary multivariable models on clinical grounds, were entered directly into a composite-iPFS Cox model, a Fine-Gray model, and a cause-specific Cox model. Continuous predictors were modeled continuously, restricted cubic splines assessed non-linearity, and time-varying coefficients addressed non-proportionality. A reduced static prediction model was evaluated as a secondary exploratory analysis with 1,000-resample bootstrap optimism correction.

**Results:**

Intracranial progression preceded death in 79 patients, whereas 25 died without previously documented progression. Median overall survival was 14.65 months (95% CI, 11.3–16.2), and median composite iPFS was 6.50 months (95% CI, 6.0–8.5). At 12 months, the cumulative incidences of intracranial progression and competing death were 63.5% and 17.3%, respectively. In complete-case analyses (*n* = 89), each doubling of neutrophil-to-lymphocyte ratio (NLR) was associated with higher hazards in the composite Cox model (HR, 1.47; 95% CI, 1.10–1.96), time-varying Fine-Gray model (sHR, 1.44; 95% CI, 1.03–2.01), and cause-specific Cox model (HR, 1.58; 95% CI, 1.14–2.19). CD4/CD8 ratio was associated with composite and cause-specific hazards but not with the Fine-Gray subdistribution hazard. The reduced static model had an optimism-corrected C-index of 0.703 and calibration slope of 0.838; calibration weakened at later horizons.

**Conclusion:**

Higher baseline NLR showed the most consistent association with both general prognosis and documented intracranial progression after GKRS. The endpoint-dependent CD4/CD8 findings and the internally validated prediction model remain exploratory and require external multicenter validation before clinical use.

## 1 Introduction

Non-small cell lung cancer (NSCLC) accounts for approximately 85% of all lung cancers and remains one of the leading causes of cancer-related mortality worldwide (1, 2). Brain metastases (BM) are among the most common distant metastatic events in advanced NSCLC, occurring in approximately 20%–40% of patients during the disease course and with higher incidence among patients harboring driver alterations such as EGFR mutations (3–6). The development of BM significantly compromises neurological function, quality of life, and overall survival. Therefore, accurate prognostic assessment and individualized management strategies remain important clinical challenges for patients with NSCLC-associated BM.

Current treatment strategies for NSCLC BM include whole-brain radiotherapy (WBRT), stereotactic radiosurgery (SRS), and surgical resection. Although WBRT provides coverage for multiple lesions, it is associated with neurocognitive toxicity. Surgical resection is generally considered for selected patients with large or symptomatic lesions, substantial mass effect, or a need for histopathological diagnosis, whereas SRS is commonly used for appropriately selected patients with limited intracranial disease (7–9). With the advent of immune checkpoint inhibitors (ICIs) and molecular targeted therapies, systemic treatment has markedly improved outcomes, yet intracranial control remains heterogeneous (10, 11). Gamma Knife radiosurgery (GKRS), as a precise and efficient SRS modality, has shown favorable local control in patients with NSCLC-BM and is widely employed. However, some patients experience early intracranial progression after GKRS, suggesting substantial interindividual differences in outcome and highlighting the need for additional prognostic biomarkers (12, 13).

Tumor immune microenvironment (TIME) has been increasingly recognized as an important determinant of tumor progression and response to anticancer therapies. Immune activation, particularly effective cytotoxic T-cell responses mediated by CD8+ T cells, may enhance radiosensitivity, whereas immunosuppressive immune states may contribute to treatment resistance and disease recurrence (14, 15). Although direct characterization of TIME usually requires tumor tissue analysis, peripheral immune-inflammatory markers obtained from routine blood tests may partially reflect systemic immune status and tumor-host interactions. Among these markers, the neutrophil-to-lymphocyte ratio (NLR) has been investigated as an accessible prognostic marker across solid tumors and in patients with NSCLC brain metastases treated with radiosurgery (16, 17). Compared with tissue-based biomarkers, peripheral immune-inflammatory parameters are minimally invasive, cost-effective, and readily available in clinical practice. However, the prognostic significance of peripheral immune-inflammatory markers, particularly lymphocyte subset-related parameters such as the CD4/CD8 ratio, in patients with NSCLC BM undergoing GKRS remains insufficiently characterized.

Although prognostic scoring systems such as the Graded Prognostic Assessment (GPA) and its molecularly updated versions have been widely used for patients with brain metastases, these models mainly rely on clinical and molecular variables, including age, performance status, extracranial disease status, number of BM, and driver alterations (18). They do not explicitly incorporate systemic immune-inflammatory markers. Therefore, evaluating peripheral immune-inflammatory indicators after adjustment for established clinical and radiosurgical factors may clarify whether these markers provide complementary prognostic information in patients with NSCLC-BM treated with GKRS.

In this retrospective cohort of patients with NSCLC brain metastases treated with GKRS, higher baseline NLR was associated with composite iPFS and documented intracranial progression after multivariable adjustment. The endpoint- and time-dependent associations of CD4/CD8 ratio and pre-GKRS oncogene-targeted therapy remain exploratory. These findings describe prognostic associations after GKRS and do not establish causal treatment effects, radiosensitivity, or treatment-predictive biomarker value. The reduced prediction model requires independent external validation before clinical use.

## 2 Patients and methods

### 2.1 Patient inclusion and data collection

This single-center retrospective cohort study was conducted at Hebei General Hospital using routinely collected clinical data. The institutional GKRS database and electronic medical records identified 121 patients with NSCLC brain metastases who underwent GKRS between July 2019 and June 2021. Of these, 17 were excluded because of previous craniotomy for brain metastases (*n* = 2), previous immune-checkpoint inhibitor therapy (*n* = 12), or insufficient follow-up information (*n* = 3). The analytic cohort therefore comprised 104 patients (Supplementary Figure S1). Eligibility criteria were: (1) histologically confirmed NSCLC; (2) radiologically confirmed brain metastases treated with GKRS within 3 months after diagnosis; (3) Eastern Cooperative Oncology Group performance status ≤2; (4) peripheral inflammatory and immune measurements obtained within 1 week before GKRS; (5) available GKRS parameters and outcome follow-up; and (6) no previous whole-brain or other intracranial radiotherapy. Insufficient follow-up was defined as inability to establish both a reliable intracranial outcome assessment date and the last-known vital status. No patient was excluded solely because follow-up was short or because an endpoint had not yet occurred.

Baseline variables were abstracted before outcome modeling and included sex, age, histological subtype, number of treated brain metastases, thoracic surgery, thoracic radiotherapy, systemic chemotherapy, and pre-GKRS oncogene-targeted therapy; peripheral inflammatory and immune markers [neutrophil-to-lymphocyte ratio (NLR), monocyte-to-lymphocyte ratio (MLR), platelet-to-lymphocyte ratio (PLR), CD4/CD8 ratio, CD3%, CD3 + CD4%, CD3 + CD8%, CD16 + CD56%, CD19%, and CD4 + CD25%]; and GKRS parameters [marginal dose, isodose fraction, mean dose, lesion volume, coverage index (CI), and selectivity index (SI)]. All blood-based variables used in the primary models were measured within 1 week before GKRS. For patients with multiple brain metastases, the treated lesion with the largest volume was selected as the representative lesion. Patient-level analyses used the volume of this lesion and its corresponding prescribed marginal dose; the number of treated brain metastases was recorded separately. Pre-GKRS oncogene-targeted therapy was defined as EGFR- or ALK-directed tyrosine kinase inhibitor treatment initiated before GKRS according to documented actionable alterations.

### 2.2 Outcomes and imaging assessment

The primary time-to-event endpoint was composite iPFS, defined as the interval from GKRS initiation to the first documented intracranial progression or death from any cause, whichever occurred first. Patients without either event would have been censored at their last evaluable follow-up; however, every patient in this cohort ultimately experienced a composite event. To distinguish intracranial failure from death due to systemic disease, documented intracranial progression was also analyzed as a separate endpoint: progression was coded as the event of interest, and death without prior documented intracranial progression was coded as a competing event. Overall survival (OS) was defined from GKRS initiation to death from any cause. Intracranial progression was determined according to RANO-BM criteria: a ≥20% increase from nadir in the sum of the longest diameters of target lesions with an absolute increase of ≥5 mm in at least one target lesion, unequivocal progression of non-target intracranial disease, or a new intracranial lesion (19).

All available contrast-enhanced brain MRI examinations were reviewed chronologically. Adjudication incorporated serial changes on gadolinium-enhanced T1-weighted, T2-FLAIR, and diffusion-weighted images, the relationship of the lesion to the previously irradiated region, neurological symptoms, corticosteroid response, and treatment history. Sustained enlargement on consecutive examinations, new nodular enhancement beyond the treated target, or a new lesion favored tumor progression; stabilization or subsequent regression without additional antitumor treatment favored treatment-related change. If conventional MRI remained equivocal, available perfusion MRI, magnetic resonance spectroscopy, or metabolic imaging was considered, followed by multidisciplinary review. Histopathological findings were used when surgical tissue was available. An indeterminate enlargement was not counted as progression until confirmed by subsequent imaging or pathology. Radiation necrosis was adjudicated using serial imaging evolution, its relationship to the irradiated region, clinical manifestations, corticosteroid response, and available advanced imaging findings (20). Because not every suspected case underwent surgery, the diagnosis was based primarily on integrated clinical and imaging evidence.

### 2.3 Statistical analysis of composite and competing-risk endpoints

Kaplan–Meier methods estimated OS and composite iPFS, and cumulative incidence functions estimated the probabilities of intracranial progression and competing death. Three complementary multivariable association models were defined before fitting the primary models on clinical grounds: (1) Cox regression for composite iPFS; (2) Fine-Gray regression for the subdistribution hazard and cumulative incidence of intracranial progression; and (3) cause-specific Cox regression for the instantaneous progression hazard among patients still alive and progression-free, with competing deaths censored (21). The adjustment set comprised the two biomarkers of primary interest (baseline NLR and CD4/CD8 ratio) and four covariates selected to represent age, molecularly selected systemic treatment exposure, the largest treated lesion, and radiosurgical dose: age, pre-GKRS oncogene-targeted therapy, largest-lesion volume, and the corresponding marginal dose. Model complexity was deliberately limited for the available event count, and selection was not based on univariable *P* values. NLR and largest-lesion volume were log2-transformed because of right-skewed distributions, so their estimates represent a doubling; age was scaled per 10-year increase and CD4/CD8 ratio per 0.5-unit increase. Primary complete-case analyses included 89 patients, 89 composite events, 67 intracranial progressions, and 22 competing deaths.

### 2.4 Functional form and proportional-hazards assessment

The proportional-hazards assumption was assessed for each covariate and globally in the composite and cause-specific Cox models using scaled Schoenfeld residuals, supplemented by likelihood-ratio tests comparing models with and without covariate-by-log-time interactions (22). Proportional subdistribution hazards in the Fine-Gray model were assessed using analogous covariate-by-centered-log-time interactions. Largest-lesion volume showed non-proportionality in both Cox models and was modeled with a time-varying coefficient. In the Fine-Gray model, pre-GKRS oncogene-targeted therapy and largest-lesion volume showed time-dependent subdistribution effects and were modeled accordingly. Time-specific HRs or sHRs were summarized at 3, 6, 12, and 18 months. Separately, restricted cubic splines with knots at the 10th, 50th, and 90th percentiles were fitted for NLR, CD4/CD8 ratio, largest-lesion volume, and corresponding marginal dose to examine functional form without imposing linearity (23).

### 2.5 Secondary exploratory prediction analysis

A reduced static Cox model for the composite iPFS event was evaluated as a secondary exploratory prediction analysis. To obtain a parsimonious model with static coefficients, largest-lesion volume was omitted because its association varied over time. The retained predictors were log2 NLR, CD4/CD8 ratio per 0.5-unit increase, pre-GKRS oncogene-targeted therapy, corresponding marginal dose, and age per 10 years. Internal validation used 1,000 bootstrap samples (24). In each replicate, the model was refitted in the bootstrap sample and evaluated both in that sample and in the original cohort; the mean performance difference was treated as optimism and subtracted from the apparent estimate. Harrell's C-index summarized overall discrimination, while time-dependent area under the curve (AUC), Brier score, calibration intercept, and calibration slope were estimated at 6, 12, and 18 months. Table 4 reports the regression coefficients, predictor coding, centering values, and baseline survival. A dynamic model retaining the time-varying largest-lesion-volume effect was examined as a sensitivity analysis.

### 2.6 Follow-up

Brain MRI, including axial gadolinium-enhanced T1-weighted, T2-FLAIR, and diffusion-weighted sequences, was scheduled 4–6 weeks after GKRS and approximately every 3 months thereafter. For all 25 patients who died without previously documented intracranial progression, the last brain MRI had been performed within 2 months before death. The last follow-up was conducted in May 2024 using medical records, outpatient visits, or telephone interviews.

### 2.7 Adverse events assessment

Treatment-related adverse events after GKRS were retrospectively assessed according to the Common Terminology Criteria for Adverse Events (CTCAE), version 5.0. Acute adverse events were defined as events occurring within 1 month after GKRS, whereas late adverse events were assessed during subsequent follow-up. Clinical symptoms, neurological examinations, medical records, outpatient follow-up notes, telephone follow-up records and follow-up MRI findings were reviewed. Radiation-induced brain edema and radiation necrosis were identified based on radiological findings and clinical assessment during follow-up. Severe adverse events were defined as CTCAE grade ≥3 toxicities.

### 2.8 General statistical procedures

Analyses were performed in R version 4.3.3 using the survival and cmprsk packages for time-to-event and competing-risk models. Continuous variables are summarized as median (IQR), and categorical variables as number (%). Effect estimates are reported with 95% confidence intervals (CIs); all tests were two-sided with *P* < 0.05 considered statistically significant. Missing values were not imputed, and analysis-specific denominators are reported: CD4/CD8 and lymphocyte-subset measurements were available for 89 patients, MLR for 98, and CI/SI for 91. Major baseline characteristics and outcomes were compared between patients with and without CD4/CD8 measurements. Crude associations of all available patient-level baseline variables with each endpoint were examined in exploratory univariable models (Supplementary Table S1), without multiplicity adjustment; their *P* values were not used for covariate selection. Sensitivity analyses added MLR or PLR, substituted CD4% or CD8% for CD4/CD8, substituted mean dose for marginal dose, omitted pre-GKRS oncogene-targeted therapy or largest-lesion volume (Supplementary Table S2), or additionally adjusted for the number of treated brain metastases (Supplementary Table S7).

### 2.9 Ethics statement

This retrospective study was approved by the Ethics Committee of Hebei General Hospital (Approval No. K-2020–770; 2020 Scientific Research Ethics Review No. 262). The study involved secondary analysis of anonymized clinical data collected during routine medical care.

## 3 Results

### 3.1 Patient characteristics

Of 121 patients screened, 17 were excluded and 104 were included in the analysis (Supplementary Figure S1). Baseline characteristics of the study cohort are summarized in Table 1. The cohort included 72 men (69.2%) and 32 women (30.8%), with a median age of 64 years (IQR, 55–68). Adenocarcinoma was present in 70 patients (67.3%), and 42 (40.4%) had initiated oncogene-targeted therapy before GKRS. Seventy-three patients (70.2%) had one treated brain metastasis, 20 (19.2%) had two, and 11 (10.6%) had three or more. Median NLR was 2.75 (IQR, 2.11–4.14), median CD4/CD8 ratio was 1.49 (IQR, 1.43–1.71), median corresponding marginal dose was 14 Gy (IQR, 13–15), and median largest-lesion volume was 2.395 cm³ (IQR, 0.946–3.803). CD4/CD8 and other lymphocyte-subset measurements were available for 89 patients. The 15 patients without CD4/CD8 data had fewer treated tumors (*P* = 0.033) and shorter OS (*P* = 0.046) than the 89 patients with available measurements; the remaining compared baseline characteristics, documented progression, and composite iPFS did not differ significantly (Supplementary Table S6).

**Table 1:** Baseline characteristics of the study cohort.

Characteristic	Value	Available *n*
Age, years	64 (55–68)	104
Sex, *n* (%)		104
Male	72 (69.2)	
Female	32 (30.8)	
Histology, *n* (%)		104
Adenocarcinoma	70 (67.3)	
Squamous cell carcinoma	32 (30.8)	
Other	2 (1.9)	
Number of treated brain metastases, *n* (%)		104
1	73 (70.2)	
2	20 (19.2)	
≥3	11 (10.6)	
Thoracic surgery, yes, *n* (%)	28 (26.9)	104
Thoracic radiotherapy, yes, *n* (%)	25 (24.0)	104
Chemotherapy, yes, *n* (%)	83 (79.8)	104
Pre-GKRS oncogene-targeted therapy, yes, *n* (%)	42 (40.4)	104
NLR	2.75 (2.11–4.14)	104
MLR	0.28 (0.17–0.36)	98
PLR	183.34 (124.96–217.01)	104
CD3, %	71.66 (67.76–73.59)	89
CD3+ CD4+, %	39.25 (37.27–44.59)	89
CD3+ CD8+, %	25.76 (25.17–26.64)	89
CD4/CD8 ratio	1.49 (1.43–1.71)	89
CD16+ CD56+, %	16.46 (14.58–19.84)	89
CD19, %	9.10 (7.64–12.34)	89
CD4+ CD25+, %	6.36 (3.70–8.90)	89
Marginal dose for largest lesion, Gy	14 (13–15)	104
Isodose fraction	0.50 (0.50–0.50)	104
Mean dose, Gy	20.1 (17.75–21.93)	104
Largest-lesion volume, cm^3^	2.395 (0.946–3.803)	104
Coverage index	0.99 (0.98–1.00)	91
Selectivity index	0.70 (0.625–0.75)	91

Continuous variables are presented as median (IQR); categorical variables as *n* (%). Largest-lesion volume and corresponding marginal dose describe the same representative treated lesion and do not represent total intracranial tumor burden.

GKRS, Gamma Knife radiosurgery; NLR, neutrophil-to-lymphocyte ratio; MLR, monocyte-to-lymphocyte ratio; PLR, platelet-to-lymphocyte ratio.

### 3.2 Survival outcomes

By the end of follow-up, all 104 patients had died. Intracranial progression was documented before death in 79 patients, whereas 25 died without prior documented intracranial progression. All 104 patients therefore experienced the composite iPFS event, and none was censored for that endpoint. Median OS was 14.65 months (95% CI, 11.3–16.2), and the estimated OS probabilities at 6, 12, and 18 months were 83.7%, 56.7%, and 33.7%, respectively (Figure 1A). Median composite iPFS was 6.50 months (95% CI, 6.0–8.5), and the corresponding probabilities of remaining free from the composite iPFS event at 6, 12, and 18 months were 59.6%, 19.2%, and 9.6%, respectively (Figure 1B). These latter estimates describe the probability of being alive without documented intracranial progression and should not be interpreted as probabilities of intracranial control alone.

**Figure 1:**
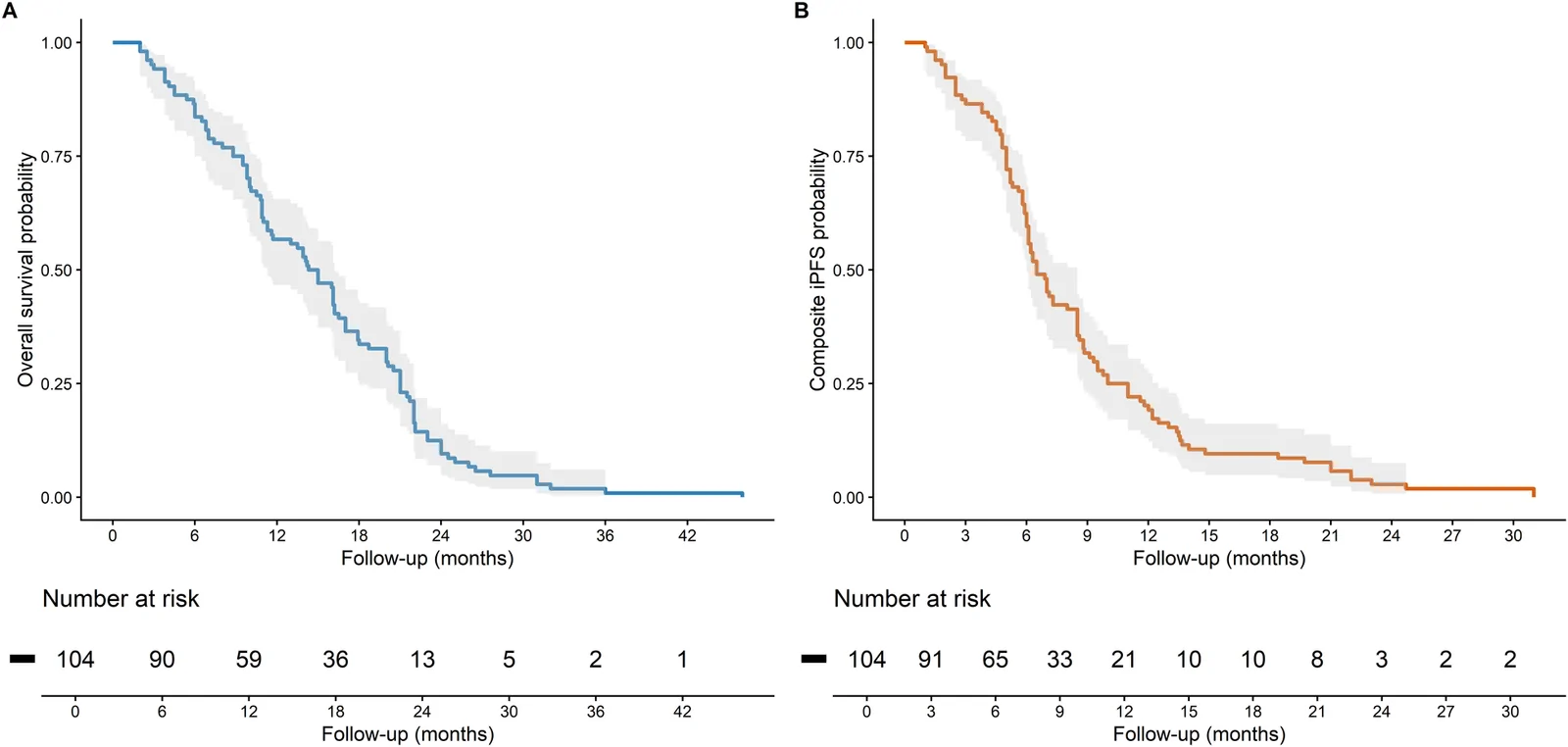
Kaplan–Meier curves for overall survival and composite intracranial progression-free survival. **(A)** Kaplan–Meier curve for overall survival. **(B)** Kaplan–Meier curve for composite intracranial progression-free survival. Numbers at risk are shown below each curve. Composite iPFS was defined as the time from GKRS initiation to intracranial progression or death from any cause, whichever occurred first. OS, overall survival; iPFS, intracranial progression-free survival; GKRS, Gamma Knife radiosurgery.

### 3.3 Cumulative incidence of intracranial progression

When death without prior progression was treated as a competing event, the cumulative incidence of documented intracranial progression was 29.8% (95% CI, 21.0–38.6) at 6 months, 63.5% (95% CI, 54.1–72.8) at 12 months, and 73.1% (95% CI, 64.4–81.7) at 18 months. The corresponding cumulative incidences of death without previously documented intracranial progression were 10.6% (95% CI, 4.6–16.5), 17.3% (95% CI, 10.0–24.6), and 17.3% (95% CI, 10.0–24.6), respectively (Figure 2). The separation of these two event types explains why the composite iPFS curve should not be used to estimate the probability of intracranial failure alone.

**Figure 2:**
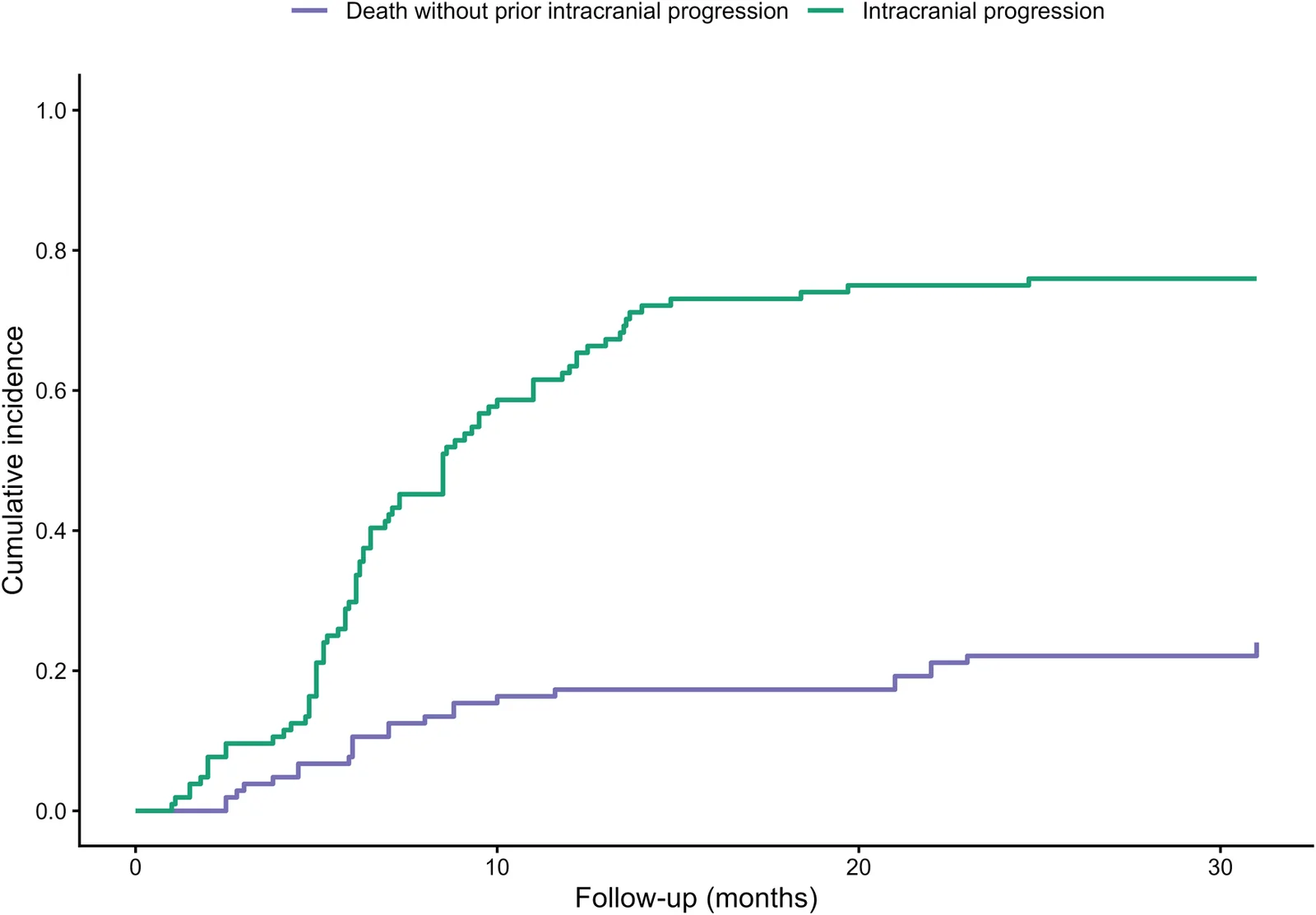
Cumulative incidence functions for intracranial progression and death without prior documented intracranial progression. Death without previously documented intracranial progression was treated as a competing event.

### 3.4 Exploratory univariable analyses

Exploratory crude models were fitted separately for all 23 patient-level baseline variables available in the final dataset and for each of the three endpoint formulations (Supplementary Table S1). Age and NLR showed nominal associations across all three analyses. Several other variables, including pre-GKRS oncogene-targeted therapy, MLR, marginal dose, mean dose, tumor volume, thoracic surgery, and CD16 + CD56%, were associated with one or two endpoint formulations. Because these estimates were unadjusted and 23 comparisons were examined without multiplicity correction, they were used to describe the data rather than to select covariates or define a significance-optimized multivariable model.

### 3.5 Multivariable association analyses

In the primary complete-case analysis (*n* = 89), higher NLR, higher CD4/CD8 ratio, older age, and absence of pre-GKRS oncogene-targeted therapy were associated with a higher hazard of the composite iPFS event after multivariable adjustment. Per doubling of NLR, the adjusted HR was 1.47 (95% CI, 1.10–1.96; *P* = 0.009). Per 0.5-unit increase in the CD4/CD8 ratio, the adjusted HR was 1.36 (95% CI, 1.03–1.80; *P* = 0.029). Pre-GKRS oncogene-targeted therapy was associated with a lower composite-event hazard (HR, 0.35; 95% CI, 0.21–0.59; *P* < 0.001), whereas each 10-year increase in age was associated with a higher hazard (HR, 1.50; 95% CI, 1.14–1.98; *P* = 0.004). The corresponding marginal dose was not associated with the composite endpoint (HR per 1 Gy, 0.91; 95% CI, 0.81–1.02; *P* = 0.115). Because largest-lesion volume had a time-varying effect, its HR per doubling was 1.38 (95% CI, 1.09–1.73; *P* = 0.007) at 3 months, 1.12 (95% CI, 0.98–1.29; *P* = 0.089) at 6 months, 0.92 (95% CI, 0.79–1.07; *P* = 0.262) at 12 months, and 0.82 (95% CI, 0.67–1.00; *P* = 0.052) at 18 months (Table 2; Figure 3A).

**Table 2:** Multivariable association models for composite iPFS and intracranial progression.

Predictor	Composite iPFS cox HR (95% CI); P	Fine-gray sHR (95% CI); P	Cause-specific cox HR (95% CI); P
NLR, per doubling	1.47 (1.10–1.96); 0.009	1.44 (1.03–2.01); 0.031	1.58 (1.14–2.19); 0.006
CD4/CD8 ratio, per 0.5	1.36 (1.03–1.80); 0.029	1.17 (0.88–1.57); 0.283	1.355 (1.004–1.830); 0.047
Pre-GKRS oncogene-targeted therapy, yes vs. no	0.35 (0.21–0.59); <0.001		0.46 (0.26–0.81); 0.007
at 3 months		0.33 (0.10–1.15); 0.082	
at 6 months		0.88 (0.47–1.63); 0.675	
at 12 months		2.30 (1.08–4.90); 0.031	
at 18 months		4.05 (1.29–12.69); 0.016	
Marginal dose for largest lesion, per Gy	0.91 (0.81–1.02); 0.115	1.01 (0.88–1.15); 0.908	0.93 (0.81–1.06); 0.271
Age, per 10 years	1.50 (1.14–1.98); 0.004	1.68 (1.24–2.27); <0.001	1.69 (1.25–2.29); <0.001
Largest-lesion volume, per doubling			
at 3 months	1.38 (1.09–1.73); 0.007	1.32 (0.99–1.75); 0.054	1.43 (1.08–1.90); 0.013
at 6 months	1.12 (0.98–1.29); 0.089	1.12 (0.95–1.31); 0.170	1.14 (0.97–1.34); 0.111
at 12 months	0.92 (0.79–1.07); 0.262	0.95 (0.79–1.13); 0.540	0.90 (0.75–1.09); 0.293
at 18 months	0.82 (0.67–1.00); 0.052	0.86 (0.66–1.11); 0.238	0.79 (0.61–1.03); 0.083

Primary complete-case models included 89 patients. Death without prior documented intracranial progression was the competing event in the Fine-Gray analysis and was censored in the cause-specific Cox analysis. Largest-lesion volume was modeled with a time-varying coefficient in all three models; pre-GKRS oncogene-targeted therapy was also time varying in the Fine-Gray model.

**Figure 3:**
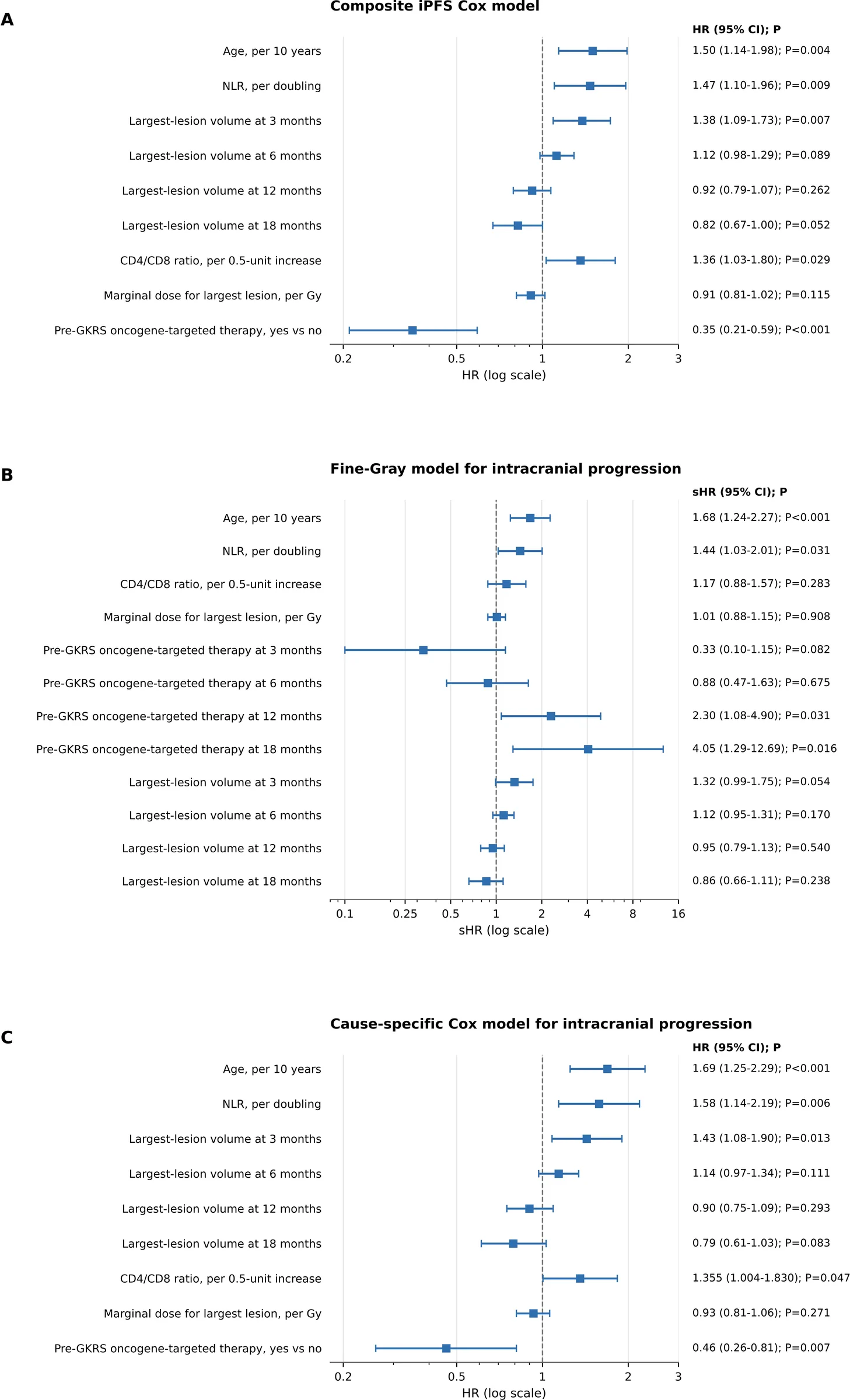
Forest plots for the three multivariable association models. **(A)** Composite iPFS Cox model. **(B)** Fine-Gray model for intracranial progression. **(C)** Cause-specific Cox model for intracranial progression. Largest-lesion-volume effects are time specific in all three models; the pre-GKRS oncogene-targeted-therapy effect is also time specific in the Fine-Gray model because of non-proportional subdistribution hazards. HR, hazard ratio; sHR, subdistribution hazard ratio.

In the Fine-Gray model, NLR (sHR per doubling, 1.44; 95% CI, 1.03–2.01; *P* = 0.031) and age (sHR per 10 years, 1.68; 95% CI, 1.24–2.27; *P* < 0.001) were associated with the subdistribution hazard of documented intracranial progression after multivariable adjustment. CD4/CD8 ratio and corresponding marginal dose were not associated with this endpoint. Pre-GKRS oncogene-targeted therapy and largest-lesion volume had time-varying subdistribution effects. The targeted-therapy sHRs were 0.33, 0.88, 2.30, and 4.05 at 3, 6, 12, and 18 months, respectively; the corresponding largest-lesion-volume sHRs per doubling were 1.32, 1.12, 0.95, and 0.86 (Table 2; Figure 3B). In the cause-specific Cox model, higher NLR (HR per doubling, 1.58; 95% CI, 1.14–2.19; *P* = 0.006), higher CD4/CD8 ratio (HR per 0.5 unit, 1.355; 95% CI, 1.004–1.830; *P* = 0.047), older age (HR per 10 years, 1.69; 95% CI, 1.25–2.29; *P* < 0.001), and absence of pre-GKRS oncogene-targeted therapy (HR for yes vs. no, 0.46; 95% CI, 0.26–0.81; *P* = 0.007) were associated with the instantaneous progression hazard after multivariable adjustment. The largest-lesion-volume association was strongest early, with HRs per doubling of 1.43, 1.14, 0.90, and 0.79 at 3, 6, 12, and 18 months, respectively (Table 2; Figure 3C).

### 3.6 Functional-form and proportional-hazards diagnostics

Restricted cubic spline analyses provided no evidence that the associations of NLR, CD4/CD8 ratio, largest-lesion volume, or corresponding marginal dose departed from linearity on their modeled scales in any endpoint model (all *P* for non-linearity >0.05; Supplementary Table S3 and Figure S2). Schoenfeld-residual and complementary log-time interaction tests showed non-proportionality for largest-lesion volume in the composite and cause-specific Cox models (Supplementary Table S4). In the Fine-Gray model, the global proportional-subdistribution-hazards test was significant (*P* = 0.012), driven by pre-GKRS oncogene-targeted therapy (*P* = 0.001) and largest-lesion volume (*P* = 0.007); these two effects were therefore modeled as time varying (Supplementary Table S5). In a sensitivity analysis additionally adjusting for number of treated tumors, this count was not associated with the composite (HR, 0.87; *P* = 0.180), Fine-Gray (sHR, 1.09; *P* = 0.460), or cause-specific endpoint (HR, 0.90; *P* = 0.340), and the NLR and CD4/CD8 estimates remained similar (Supplementary Table S7).

### 3.7 Secondary exploratory prediction analysis

The reduced static prediction model satisfied the proportional-hazards assumption (global Schoenfeld *P* = 0.484). Its apparent Harrell C-index was 0.718; after 1,000 bootstrap resamples, the optimism-corrected C-index was 0.703 and the overall calibration slope was 0.838. The optimism-corrected AUCs were 0.783, 0.773, and 0.823 at 6, 12, and 18 months, respectively; the corresponding corrected calibration slopes were 1.087, 0.661, and 0.443 (Table 3; Figure 4). Curves in Figure 4 show apparent performance, whereas the numerical annotations report bootstrap optimism-corrected metrics. Thus, discrimination was moderate within the development cohort, but calibration became less stable at later horizons, particularly at 18 months. Full model coefficients, coding, and baseline survival are reported in Table 4. The dynamic sensitivity model yielded a corrected C-index of 0.708 (Supplementary Table S8 and Figure S3). Neither model was externally validated.

**Table 3:** Bootstrap-corrected performance of the simplified static prediction model.

Metric	Horizon	Apparent	Optimism-corrected
Harrell C-index	Overall	0.718	0.703
Overall calibration slope	Overall	1.000	0.838
Time-dependent AUC	6 months	0.799	0.783
Brier score	6 months	0.174	0.187
Calibration slope	6 months	1.284	1.087
Time-dependent AUC	12 months	0.789	0.773
Brier score	12 months	0.133	0.144
Calibration slope	12 months	0.749	0.661
Time-dependent AUC	18 months	0.833	0.823
Brier score	18 months	0.074	0.080
Calibration slope	18 months	0.693	0.443

All optimism corrections were based on 1,000 successful bootstrap resamples. The simplified model included log2 NLR, CD4/CD8 ratio, pre-GKRS oncogene-targeted therapy, corresponding marginal dose, and age; largest-lesion volume was deliberately omitted to construct a parsimonious static model because its effect was time varying. Curves in Figure 4 show apparent performance, while annotations report optimism-corrected metrics.

**Figure 4:**
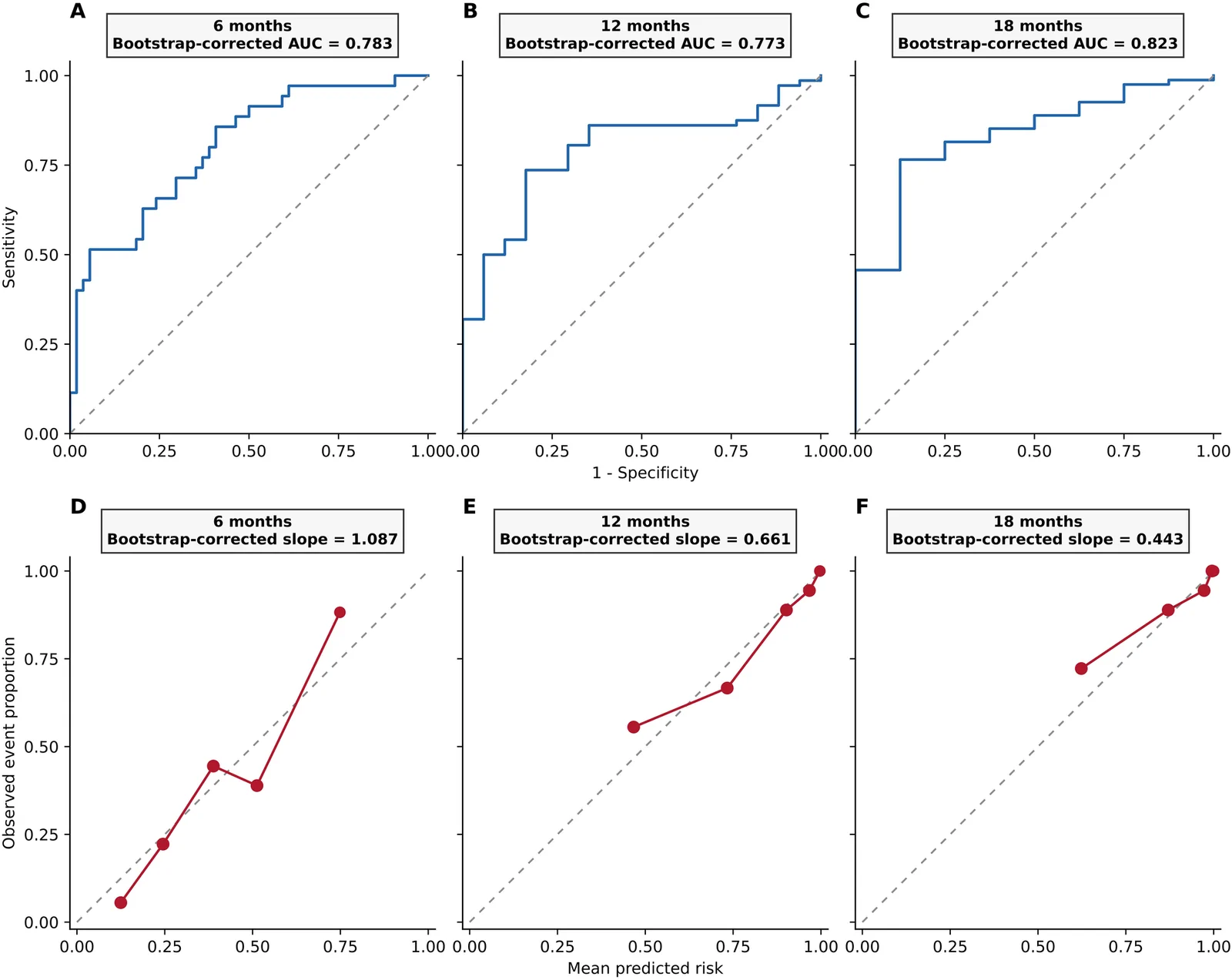
Internal validation of the simplified static prediction model. **(A–C)** Apparent time-dependent receiver operating characteristic curves at 6, 12, and 18 months; numerical annotations report bootstrap optimism-corrected AUCs. **(D–F)** Apparent grouped calibration plots at 6, 12, and 18 months; numerical annotations report bootstrap optimism-corrected calibration slopes. Curves show apparent performance; numerical annotations report bootstrap optimism-corrected metrics based on 1,000 resamples. AUC, area under the curve.

**Table 4:** Reproducible specification of the simplified static prediction model.

Predictor	Coding	Centering value	Coefficient
NLR	log2(NLR)	1.56106	0.41747
CD4/CD8 ratio	ratio/0.5	3.15062	0.24824
Pre-GKRS oncogene-targeted therapy	yes = 1; no = 0	0.40449	−1.05059
Marginal dose for largest lesion	Gy	13.85393	−0.10848
Age	years/10	6.26292	0.33752
Baseline survival	6 months		0.64477
Baseline survival	12 months		0.12065
Baseline survival	18 months		0.03561

For a patient, LP = Σβj(Xj − centerj), and the predicted composite-iPFS event risk at time t is 1 − S0(t)^exp(LP)^. Coefficients were estimated in the 89 complete cases; the table reports baseline survival for the mean covariate profile.

### 3.8 Adverse events and safety

All patients tolerated GKRS without CTCAE grade ≥3 toxicity. Twenty-six patients (25.0%) experienced grade 1–2 acute symptoms, including headache, nausea, or vomiting, which resolved with symptomatic treatment. During later follow-up, eight patients (7.7%) developed radiation-associated cerebral edema that improved with corticosteroid or supportive treatment. Four patients (3.8%) were adjudicated as having radiation necrosis; none developed severe neurological deficits.

## 4 Discussion

The present study evaluated two related but clinically distinct outcomes after GKRS. Composite iPFS describes the time until either intracranial progression or death and therefore reflects both intracranial disease control and the patient's overall disease course. In contrast, the cumulative-incidence, Fine-Gray, and cause-specific analyses focus on documented intracranial progression while accounting for patients who die before progression can be observed. Across these complementary analyses, higher baseline NLR was the most consistent immune-inflammatory correlate of adverse outcome. The associations of CD4/CD8 ratio and pre-GKRS oncogene-targeted therapy varied according to endpoint definition, and largest-lesion volume showed a time-dependent association concentrated early after treatment. The findings characterize prognosis after GKRS and do not evaluate treatment-effect prediction.

NLR is inexpensive, reproducible from routine blood counts, and has repeatedly been associated with adverse outcomes across solid tumors, in patients receiving radiotherapy, and in patients with NSCLC brain metastases treated with radiosurgery (16, 17, 25). Our analysis adds a narrower observation: when NLR was retained as a continuous variable, each doubling was associated not only with the composite endpoint but also with both the time-varying Fine-Gray model and the cause-specific hazard of intracranial progression. The concordant estimates across complementary endpoint models support NLR as the most consistent immune-inflammatory association in this cohort. Even so, NLR is not specific to the brain or to radiosensitivity. It can reflect tumor burden, infection, corticosteroid exposure, nutritional status, and systemic inflammatory activity. Its association with composite iPFS may therefore partly represent general prognosis, whereas the competing-risk results provide more direct, although still observational, support for an association with intracranial progression. Biologically, neutrophils may promote tumor growth through angiogenic signaling, extracellular-trap formation, and suppression of lymphocyte-mediated antitumor activity (26, 27). These mechanisms are plausible explanations but were not directly measured in this cohort, so mechanistic causality cannot be inferred.

The sensitivity analyses further qualify the NLR finding. Its estimated effect remained above 1.0 in every alternative adjustment set and remained statistically significant in every Fine-Gray and cause-specific analysis. In the composite model, however, the confidence interval crossed 1.0 after MLR or PLR was added. Because these ratios share a lymphocyte denominator and represent overlapping inflammatory pathways, simultaneous inclusion can increase collinearity and divide a common signal among correlated variables. The result should therefore not be framed as evidence that NLR is biologically unique or superior to every other blood marker. Rather, NLR appears to be the most stable representative of a broader systemic immune-inflammatory state in the available dataset. The absence of multiplicity correction in the exploratory univariable screen and the incomplete availability of some immune variables reinforce the need for confirmation in a prospectively specified external cohort.

The CD4/CD8 ratio was associated with the composite hazard and reached borderline statistical significance in the cause-specific model, but it was not associated with the Fine-Gray subdistribution hazard and was less stable across alternative adjustment sets. This endpoint dependence differs from the more consistent NLR pattern and is the principal reason that the CD4/CD8 result is described as exploratory. A higher peripheral CD4/CD8 ratio could arise from relative depletion of CD8+ T cells, expansion of CD4+ T-cell subsets, or both. CD8+ T cells are important cytotoxic effectors of antitumor immunity, whereas CD4+ T cells comprise functionally diverse populations, including conventional helper and regulatory subsets, which can exert different and sometimes opposing effects on antitumor immunity (28–30). A single peripheral ratio cannot distinguish these subsets or establish the immune composition within brain metastases. In addition, CD4/CD8 values were missing for 15 patients, reducing precision and creating potential complete-case selection. The present finding is therefore best viewed as a hypothesis for studies incorporating serial immune phenotyping, treatment exposures such as corticosteroids, and paired systemic and intracranial immune measurements.

Pre-GKRS oncogene-targeted therapy represented both actionable EGFR/ALK molecular status and actual treatment exposure. It was associated with lower composite and cause-specific hazards, whereas its Fine-Gray subdistribution effect varied over time and changed direction at later horizons. Treatment was limited to a molecularly selected subgroup, was not randomized, and individual drug, duration, adherence, post-GKRS therapy, and extracranial response could not be fully modeled. CNS-active EGFR and ALK inhibitors have demonstrated intracranial activity in appropriately selected NSCLC populations (31–34), but the present data do not separate drug exposure from tumor biology, survival-related observation time, or other treatment differences and do not assess synergy with GKRS. Pre-GKRS oncogene-targeted therapy was therefore treated as an adjusted clinical correlate in a molecularly selected subgroup.

Restricted cubic spline analyses did not identify significant non-linear associations of NLR, CD4/CD8 ratio, corresponding marginal dose, or largest-lesion volume with the study outcomes. Nevertheless, the modest sample size limited the ability to detect complex relationships at distributional extremes. Largest-lesion volume showed time-varying associations, with higher early hazards that weakened thereafter. Previous radiosurgical studies support the relevance of lesion volume and prescription dose for local control and clinical outcomes (35, 36). Documented intracranial progression was a patient-level endpoint that could result from failure of any treated lesion, non-target intracranial disease, or a new lesion, whereas volume and dose described only the largest treated lesion. These measures characterize the largest lesion rather than complete intracranial tumor burden or lesion-specific local control across all metastases. The sensitivity analysis including the number of treated tumors did not materially change the biomarker estimates; complete lesion-level outcome data were unavailable.

The secondary prediction analysis evaluated the measured variables as a potential basis for future prognostic modeling. Existing GPA-based instruments summarize established clinical and molecular prognosis in patients with brain metastases (18), whereas the reduced model incorporated immune-inflammatory and treatment variables. Bootstrap correction reduced the C-index from 0.718 to 0.703 and produced an overall calibration slope of 0.838. Calibration slopes declined at 12 and 18 months, indicating overly extreme and unstable later-horizon predictions. Clinical application for treatment decisions, risk thresholds, or MRI-surveillance schedules requires independent validation and comparison with established systems. No grade ≥3 toxicity was observed, and four cases were classified as radiation necrosis, a recognized delayed complication of SRS (9, 20); because adjudication relied largely on clinical and imaging evolution, some misclassification remains possible.

## 5 Limitations

Several limitations should be considered. First, this was a relatively small single-center retrospective cohort. CD4/CD8 measurements were unavailable for 15 patients. Those patients had fewer treated tumors and shorter OS than complete cases, indicating potential complete-case selection, although most other compared characteristics and outcomes did not differ significantly. Missing values were not imputed, and residual confounding and limited precision remain possible. Patient-level ECOG categories, complete molecular-driver status, detailed longitudinal systemic treatment, and extracranial progression were unavailable in the final analysis dataset. Second, intracranial progression and treatment-related imaging changes were adjudicated retrospectively, and advanced imaging and histopathological confirmation were not uniformly available. However, all 25 patients who died without previously documented progression underwent brain MRI within 2 months before death. Third, the largest treated lesion and its corresponding marginal dose were used as patient-level summaries, whereas the endpoint included progression anywhere in the brain; lesion-level local control was therefore not evaluated. Although the number-of-treated-tumors sensitivity analysis did not materially alter the biomarker estimates, it did not resolve this analytic-level limitation. Finally, time-varying estimates were imprecise at later horizons, and bootstrap internal validation cannot establish transportability. Multicenter external validation should incorporate lesion-level outcomes, detailed molecular and treatment data, standardized imaging assessment, and comparison with established prognostic indices.

## 6 Conclusion

In this retrospective cohort of patients with NSCLC brain metastases treated with GKRS, higher baseline NLR was consistently associated with the composite endpoint and with documented intracranial progression in competing-risk and cause-specific analyses. CD4/CD8 ratio and pre-GKRS oncogene-targeted therapy showed endpoint-dependent associations and should remain exploratory. Modeling continuous predictors, competing death, and the time-varying effect of tumor volume allowed clinically interpretable assessment of these associations. The reduced prediction model showed moderate optimism-corrected discrimination but inadequate later-horizon calibration and must not be used clinically before independent external validation.

## Data Availability

The data analyzed in this study is subject to the following licenses/restrictions: The dataset contains clinical information derived from medical records of patients with NSCLC brain metastases and is subject to privacy protection and ethical restrictions. The data are not publicly available because they contain potentially identifiable patient information. De-identified data may be available from the corresponding author on reasonable request and with approval from the institution/ethics committee. Requests to access the dataset can be made by contacting the corresponding author, Jing Zhao, via zzzjmail@163.com.
