# Supplementary material for: Peripheral immune-inflammatory markers associated with intracranial progression-free survival after Gamma Knife radiosurgery in patients with non-small cell lung cancer brain metastases: a retrospective cohort study
Source: Front Med (Lausanne). 2026 Oct 13;13:1941822. doi: 10.3389/fmed.2026.1941822 (PMC13710305; doi:10.3389/fmed.2026.1941822)
Supplement: Supplementary file 1 [file Datasheet1.pdf]

Supplementary Material

Manuscript ID 1941822

Peripheral immune-inflammatory markers associated with intracranial progression-free survival after Gamma Knife radiosurgery in patients with non-small cell lung cancer brain metastases: a retrospective cohort study

Supplementary Table S1 Exploratory univariable associations of available baseline variables with study endpoints

Variable	Analysis scale	Available n	Composite iPFS Cox HR (95% CI); P	Fine-Gray sHR (95% CI); P	Cause-specific Cox HR (95% CI); P
Sex	Male vs female	104	1.20 (0.78-1.84); 0.404	1.51 (0.92-2.48); 0.102	1.46 (0.89-2.42); 0.137
Age	Per 10-year increase	104	1.74 (1.38-2.20); <0.001	1.55 (1.22-1.98); <0.001	1.91 (1.47-2.49); <0.001
Histology	Adenocarcinoma vs other	104	0.73 (0.48-1.11); 0.142	1.09 (0.67-1.77); 0.732	0.80 (0.49-1.30); 0.364
Thoracic surgery	Yes vs no	104	0.80 (0.50-1.26); 0.333	1.85 (1.15-2.96); 0.011	1.21 (0.75-1.95); 0.430
Thoracic radiotherapy	Yes vs no	104	1.04 (0.66-1.65); 0.853	1.00 (0.59-1.69); 0.995	0.96 (0.56-1.64); 0.879
Chemotherapy	Yes vs no	104	1.41 (0.87-2.29); 0.165	1.35 (0.77-2.37); 0.297	1.61 (0.91-2.84); 0.099
Pre-GKRS oncogene-targeted therapy	Yes vs no	104	0.38 (0.25-0.58); <0.001	0.82 (0.52-1.28); 0.377	0.44 (0.27-0.70); <0.001
NLR	Per doubling	104	1.48 (1.13-1.95); 0.005	1.42 (1.05-1.92); 0.023	1.59 (1.17-2.15); 0.003
MLR	Per doubling	98	1.59 (1.18-2.14); 0.003	1.12 (0.82-1.53); 0.489	1.47 (1.04-2.06); 0.027
PLR	Per doubling	104	1.30 (0.95-1.80); 0.103	1.15 (0.83-1.59); 0.412	1.27 (0.89-1.83); 0.191
CD3, %	Per 10-percentage-point increase	89	0.94 (0.63-1.42); 0.782	0.98 (0.66-1.47); 0.931	0.96 (0.60-1.52); 0.861
CD3+CD4+, %	Per 10-percentage-point increase	89	1.33 (0.96-1.84); 0.087	1.09 (0.77-1.53); 0.627	1.33 (0.91-1.92); 0.137
CD3+CD8+, %	Per 10-percentage-point increase	89	0.96 (0.61-1.50); 0.850	0.85 (0.51-1.40); 0.511	0.87 (0.49-1.54); 0.636
CD4/CD8 ratio	Per 0.5-unit increase	89	1.26 (1.00-1.59); 0.054	1.13 (0.86-1.47); 0.379	1.28 (0.99-1.67); 0.064
CD16+CD56+, %	Per 10-percentage-point increase	89	0.94 (0.64-1.37); 0.735	0.59 (0.38-0.93); 0.022	0.69 (0.43-1.12); 0.133
CD19, %	Per 10-percentage-point increase	89	0.85 (0.51-1.41); 0.527	2.10 (1.31-3.36); 0.002	1.39 (0.83-2.35); 0.213
CD4+CD25+, %	Per 10-percentage-point increase	89	1.47 (0.72-3.00); 0.288	1.73 (0.79-3.76); 0.169	1.69 (0.75-3.78); 0.205
Corresponding marginal dose	Per 1-Gy increase	104	0.88 (0.80-0.97); 0.008	0.94 (0.84-1.06); 0.308	0.88 (0.79-0.98); 0.025
Isodose fraction	Per 0.10 increase	104	1.03 (0.77-1.39); 0.831	0.82 (0.59-1.14); 0.243	0.89 (0.62-1.29); 0.548
Mean dose	Per 1-Gy increase	104	0.92 (0.87-0.98); 0.011	1.00 (0.94-1.07); 0.992	0.95 (0.89-1.02); 0.159
Largest-lesion volume	Per doubling	104	1.22 (1.09-1.36); <0.001	1.11 (0.99-1.26); 0.083	1.23 (1.08-1.41); 0.002
Coverage index	Per 0.10 increase	91	1.00 (0.41-2.44); 0.995	1.95 (0.61-6.20); 0.257	1.32 (0.46-3.84); 0.608
Selectivity index	Per 0.10 increase	91	1.04 (0.83-1.29); 0.745	0.93 (0.72-1.20); 0.592	1.01 (0.79-1.29); 0.927
Number of treated tumors	Per one-tumor increase	104	1.01 (0.84-1.22); 0.916	1.06 (0.86-1.30); 0.575	1.02 (0.84-1.26); 0.812

Crude exploratory associations; no multiplicity correction was applied, and these P values were not used to choose variables for the primary multivariable models.

Supplementary Table S2 Sensitivity of NLR and CD4/CD8 estimates to alternative covariate adjustment sets

Sensitivity model	n	NLR: Composite	CD4/CD8: Composite	NLR: Fine-Gray	CD4/CD8: Fine-Gray	NLR: Cause-specific	CD4/CD8: Cause-specific
Primary six-covariate model	89	1.47 (1.10-1.96); 0.009	1.36 (1.03-1.80); 0.029	1.44 (1.03-2.01); 0.031	1.17 (0.88-1.57); 0.283	1.58 (1.14-2.19); 0.006	1.355 (1.004-1.830); 0.047
Add MLR	85	1.36 (0.97-1.90); 0.077	1.40 (1.04-1.88); 0.024	1.51 (1.03-2.20); 0.034	1.10 (0.82-1.47); 0.541	1.49 (1.02-2.17); 0.037	1.38 (1.00-1.90); 0.047
Add PLR	89	1.39 (0.90-2.15); 0.134	1.35 (1.02-1.79); 0.036	1.72 (1.07-2.78); 0.025	1.14 (0.85-1.53); 0.367	1.71 (1.06-2.78); 0.029	1.37 (1.01-1.87); 0.043
CD4% substituted for CD4/CD8	89	1.40 (1.05-1.87); 0.021	Not included	1.48 (1.06-2.08); 0.022	Not included	1.51 (1.10-2.07); 0.012	Not included
CD8% substituted for CD4/CD8	89	1.41 (1.05-1.89); 0.021	Not included	1.47 (1.05-2.04); 0.023	Not included	1.50 (1.08-2.09); 0.015	Not included
Mean dose substituted for marginal dose	89	1.41 (1.06-1.86); 0.017	1.34 (1.02-1.76); 0.036	1.51 (1.08-2.11); 0.016	1.12 (0.85-1.49); 0.421	1.52 (1.11-2.08); 0.009	1.34 (1.00-1.80); 0.052
Targeted therapy omitted	89	1.47 (1.09-1.98); 0.011	1.27 (0.98-1.65); 0.066	1.49 (1.07-2.08); 0.020	1.12 (0.84-1.48); 0.448	1.59 (1.14-2.22); 0.006	1.29 (0.97-1.71); 0.085
Tumor volume omitted	89	1.52 (1.14-2.03); 0.005	1.28 (0.96-1.71); 0.090	1.55 (1.12-2.15); 0.009	1.10 (0.83-1.46); 0.488	1.69 (1.22-2.33); 0.001	1.30 (0.95-1.76); 0.099
Additionally adjusted for number of treated tumors	89	1.44 (1.07-1.92); 0.015	1.38 (1.04-1.82); 0.025	1.46 (1.05-2.04); 0.026	1.16 (0.87-1.56); 0.310	1.55 (1.12-2.15); 0.009	1.37 (1.01-1.85); 0.041

Values are HR or sHR (95% CI); P value. Models retaining largest-lesion volume used its time-varying effect where required. The Fine-Gray primary and tumor-count sensitivity rows use the time-varying Fine-Gray specification.

Supplementary Table S3 Restricted cubic spline tests

Endpoint	Predictor	n	Target events	P overall	P non-linearity
Cause-specific Cox	CD4/CD8 ratio, per 0.5-unit increase	89	67	0.279	0.702
Cause-specific Cox	NLR, per doubling	89	67	0.014	0.354
Cause-specific Cox	Largest-lesion volume, per doubling	89	67	0.645	0.783
Cause-specific Cox	Marginal dose for largest lesion, per Gy	89	67	0.252	0.274
Composite iPFS Cox	CD4/CD8 ratio, per 0.5-unit increase	89	89	0.059	0.078
Composite iPFS Cox	NLR, per doubling	89	89	0.036	0.480
Composite iPFS Cox	Largest-lesion volume, per doubling	89	89	0.598	0.529
Composite iPFS Cox	Marginal dose for largest lesion, per Gy	89	89	0.220	0.569
Fine-Gray	CD4/CD8 ratio, per 0.5-unit increase	89	67	0.711	0.442
Fine-Gray	NLR, per doubling	89	67	0.012	0.207
Fine-Gray	Largest-lesion volume, per doubling	89	67	0.708	0.786
Fine-Gray	Marginal dose for largest lesion, per Gy	89	67	0.486	0.329

Knots were placed at the 10th, 50th, and 90th percentiles. P non-linearity tests the non-linear component.

Supplementary Table S4 Proportional-hazards tests for the Cox models

Endpoint	Term	Chi-square	df	P value
Composite iPFS	NLR, per doubling	0.004	1	0.950
Composite iPFS	CD4/CD8 ratio, per 0.5-unit increase	0.141	1	0.707
Composite iPFS	Pre-GKRS oncogene-targeted therapy	1.153	1	0.283
Composite iPFS	Marginal dose for largest lesion, per Gy	1.149	1	0.284
Composite iPFS	Largest-lesion volume, per doubling	8.205	1	0.004
Composite iPFS	Age, per 10 years	3.290	1	0.070
Composite iPFS	GLOBAL	11.430	6	0.076
Cause-specific intracranial progression	NLR, per doubling	0.020	1	0.889
Cause-specific intracranial progression	CD4/CD8 ratio, per 0.5-unit increase	0.378	1	0.539
Cause-specific intracranial progression	Pre-GKRS oncogene-targeted therapy	0.269	1	0.604
Cause-specific intracranial progression	Marginal dose for largest lesion, per Gy	0.631	1	0.427
Cause-specific intracranial progression	Largest-lesion volume, per doubling	5.993	1	0.014
Cause-specific intracranial progression	Age, per 10 years	1.834	1	0.176
Cause-specific intracranial progression	GLOBAL	8.493	6	0.204

Scaled Schoenfeld residual tests are shown for the initial Cox specifications.

Supplementary Table S5 Proportional subdistribution-hazards tests for the Fine-Gray model

Term	Chi-square	df	P value
NLR, per doubling	0.447	1	0.504
CD4/CD8 ratio, per 0.5-unit increase	0.048	1	0.827
Pre-GKRS oncogene-targeted therapy	10.231	1	0.001
Marginal dose for largest lesion, per Gy	3.168	1	0.075
Largest-lesion volume, per doubling	7.256	1	0.007
Age, per 10 years	1.813	1	0.178
GLOBAL	16.246	6	0.012

Likelihood-ratio tests compare the proportional model with models containing covariate-by-centered-log-time interactions. Targeted therapy and largest-lesion volume were modeled with time-varying effects in the final Fine-Gray model.

Supplementary Table S6 Comparison of patients with and without available CD4/CD8 measurements

Characteristic	Type	CD4/CD8 available (n=89)	CD4/CD8 missing (n=15)	P value
Age, years	Continuous	64.00 (55.00-68.00)	65.00 (56.00-70.50)	0.7283
NLR	Continuous	2.72 (2.01-3.99)	3.78 (2.64-4.23)	0.0895
MLR	Continuous	0.27 (0.16-0.36)	0.30 (0.24-0.34)	0.3705
PLR	Continuous	176.68 (123.95-209.78)	205.62 (143.43-242.36)	0.087
Marginal dose, Gy	Continuous	14.00 (13.00-15.00)	14.00 (12.00-15.00)	0.7332
Mean dose, Gy	Continuous	20.20 (18.20-22.00)	19.90 (17.30-21.05)	0.6907
Largest-lesion volume, cm3	Continuous	2.39 (0.95-3.60)	2.42 (1.16-4.91)	0.4935
Number of treated brain metastases	Continuous	1.00 (1.00-2.00)	1.00 (1.00-1.00)	0.0329
Male sex	Categorical	61/89 (68.5%)	11/15 (73.3%)	1.0
Adenocarcinoma	Categorical	61/89 (68.5%)	9/15 (60.0%)	0.5583
Thoracic surgery	Categorical	26/89 (29.2%)	2/15 (13.3%)	0.3446
Thoracic radiotherapy	Categorical	19/89 (21.3%)	6/15 (40.0%)	0.1871
Chemotherapy	Categorical	72/89 (80.9%)	11/15 (73.3%)	0.4972
Pre-GKRS oncogene-targeted therapy	Categorical	36/89 (40.4%)	6/15 (40.0%)	1.0
Documented intracranial progression	Categorical	67/89 (75.3%)	12/15 (80.0%)	1.0
Composite iPFS, months	Time-to-event	6.50 (5.00-10.00)	6.30 (2.65-10.32)	0.9465
Overall survival, months	Time-to-event	16.10 (9.80-21.00)	13.00 (3.95-15.50)	0.0464

Continuous variables were compared using the Mann-Whitney U test, categorical variables using Fisher’s exact test, and time-to-event outcomes using log-rank tests.

Supplementary Table S7 Sensitivity analysis additionally adjusting for number of treated tumors

Endpoint	n	Predictor	Effect (95% CI); P
Composite iPFS Cox	89	NLR, per doubling	1.44 (1.07-1.92); 0.015
Composite iPFS Cox	89	CD4/CD8 ratio, per 0.5-unit increase	1.38 (1.04-1.82); 0.025
Composite iPFS Cox	89	Number of treated tumors, per one-tumor increase	0.87 (0.71-1.07); 0.180
Cause-specific Cox	89	NLR, per doubling	1.55 (1.12-2.15); 0.009
Cause-specific Cox	89	CD4/CD8 ratio, per 0.5-unit increase	1.37 (1.01-1.85); 0.041
Cause-specific Cox	89	Number of treated tumors, per one-tumor increase	0.90 (0.71-1.12); 0.340
Fine-Gray with time-varying pre-GKRS oncogene-targeted therapy and largest-lesion volume	89	NLR, per doubling	1.46 (1.05-2.04); 0.026
Fine-Gray with time-varying pre-GKRS oncogene-targeted therapy and largest-lesion volume	89	CD4/CD8 ratio, per 0.5-unit increase	1.16 (0.87-1.56); 0.310
Fine-Gray with time-varying pre-GKRS oncogene-targeted therapy and largest-lesion volume	89	Number of treated tumors, per one-tumor increase	1.09 (0.87-1.36); 0.460

The Cox models retained the time-varying largest-lesion-volume effect. The Fine-Gray model retained time-varying effects for pre-GKRS oncogene-targeted therapy and largest-lesion volume.

Supplementary Table S8 Bootstrap-corrected performance of the dynamic sensitivity prediction model

Metric	Horizon, months	Apparent	Optimism-corrected	Successful resamples
Dynamic C-index (Antolini)		0.730	0.708	1000
Time-dependent AUC	6.0	0.808	0.789	1000
Brier score	6.0	0.168	0.183	1000
Calibration intercept	6.0	0.043	-0.064	1000
Calibration slope	6.0	1.098	0.909	1000
Time-dependent AUC	12.0	0.792	0.771	1000
Brier score	12.0	0.133	0.145	1000
Calibration intercept	12.0	0.106	0.123	1000
Calibration slope	12.0	0.706	0.614	1000
Time-dependent AUC	18.0	0.840	0.818	1000
Brier score	18.0	0.075	0.083	1000
Calibration intercept	18.0	0.292	0.348	1000
Calibration slope	18.0	0.670	0.509	1000

The dynamic model retained the time-varying largest-lesion-volume effect and was evaluated with 1,000 bootstrap resamples.

Supplementary Figure S1 Patient selection flow diagram

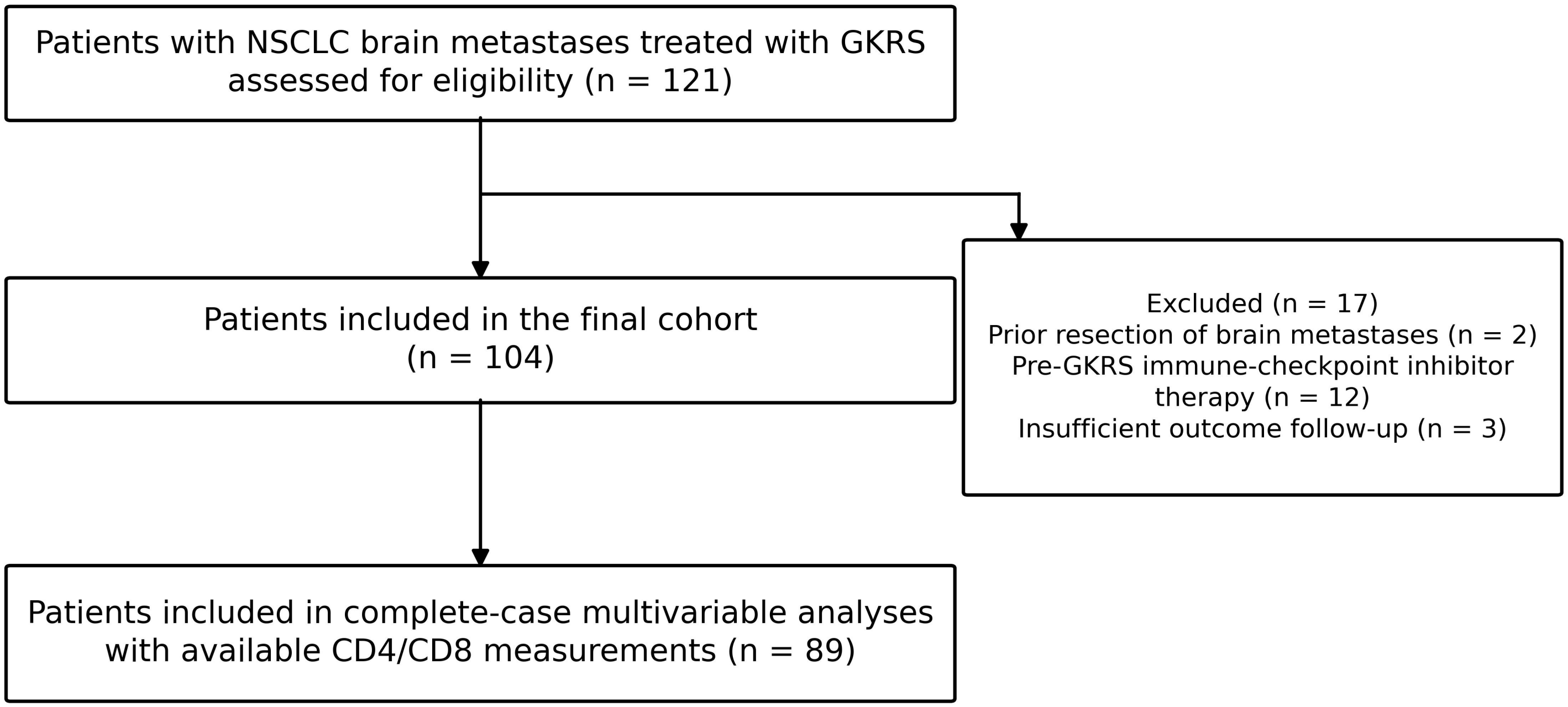

Of 121 patients screened, 17 were excluded (previous craniotomy for brain metastases, n=2; prior immune-checkpoint inhibitor treatment, n=12; insufficient follow-up to establish reliable intracranial outcome assessment and last-known vital status, n=3), leaving 104 patients for analysis. No patient was excluded solely because follow-up was short or no endpoint event had occurred.

Supplementary Figure S2 Restricted cubic spline analyses for NLR and CD4/CD8 ratio in the composite iPFS model

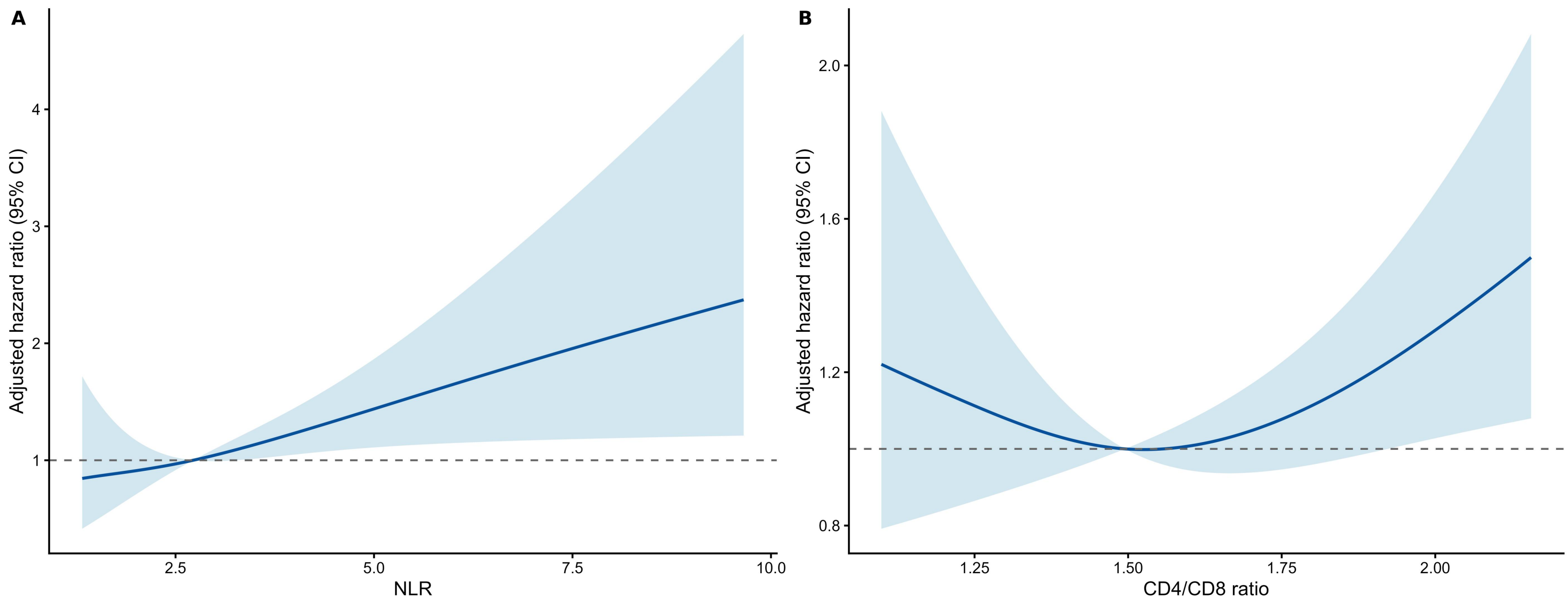

Solid lines show estimated hazard ratios and shaded areas show 95% confidence intervals. Knots were placed at the 10th, 50th, and 90th percentiles.

Supplementary Figure S3 Internal validation of the dynamic sensitivity prediction model

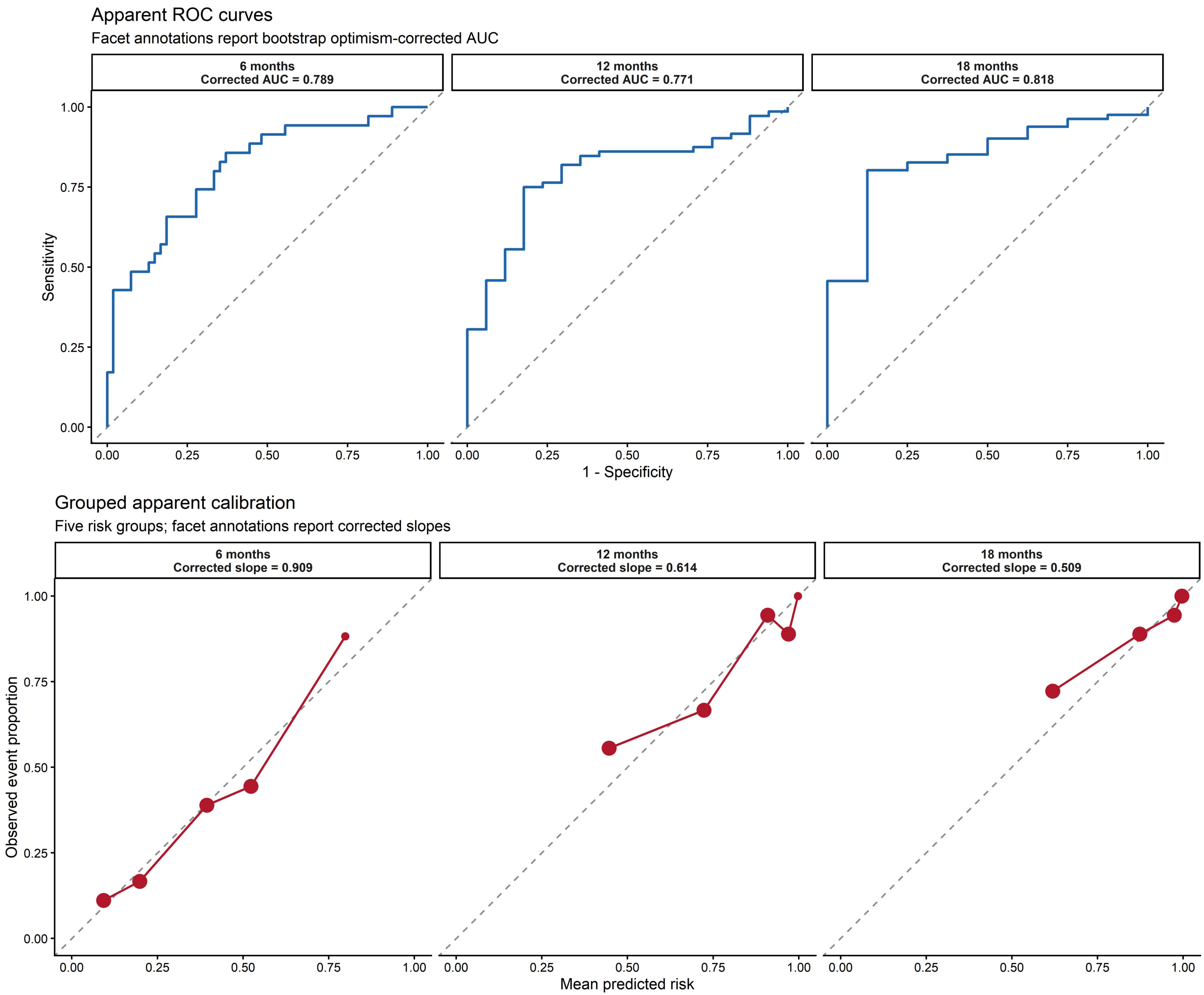

The dynamic model incorporated the time-varying largest-lesion-volume effect and was evaluated with 1,000 bootstrap resamples. Curves are apparent estimates; facet annotations report bootstrap optimism-corrected performance.
